# Analysis of virulence factors and antibiotic resistance genes in group B streptococcus from clinical samples

**DOI:** 10.1186/s12879-021-05820-6

**Published:** 2021-01-28

**Authors:** Raymond Mudzana, Rooyen T. Mavenyengwa, Muchaneta Gudza-Mugabe

**Affiliations:** 1 Department of Medical Microbiology, National Polio Laboratory, University of Zimbabwe College of Health Sciences, P. O. Box A178, Avondale, Harare, Zimbabwe; 2 Faculty of Health Sciences, Institute of Infectious Diseases and Molecular Medicine, University of Cape Town, Room No. 3.22 Falmouth Building, Anzio Road, Observatory, Cape Town, 7925 South Africa

**Keywords:** Group B streptococcus, Virulence genes, Antibiotic resistance genes, PCR, Zimbabwe

## Abstract

**Background:**

*Streptococcus agalacticae* (Group B *Streptococcus*, GBS) is one of the most important causative agents of serious infections among neonates. This study was carried out to identify antibiotic resistance and virulence genes associated with GBS isolated from pregnant women.

**Methods:**

A total of 43 GBS isolates were obtained from 420 vaginal samples collected from HIV positive and negative women who were 13–35 weeks pregnant attending Antenatal Care at Chitungwiza and Harare Central Hospitals in Zimbabwe. Identification tests of GBS isolates was done using standard bacteriological methods and molecular identification testing. Antibiotic susceptibility testing was done using the modified Kirby-Bauer method and E-test strips. The boiling method was used to extract DNA and Polymerase Chain Reaction (PCR) was used to screen for 13 genes. Data was fed into SPSS 24.0.

**Results:**

Nine distinct virulence gene profiles were identified and *hly-scpB-bca-rib* 37.2% (16/43) was common. The virulence genes identified were namely *hly* 97.8% (42/43), *scpB* 90.1% (39/43), *bca* 86.0% (37/43), *rib* 69.8% (30/43) and *bac* 11.6% (5/43). High resistance to tetracycline 97.7% (42/43) was reported followed by 72.1% (31/43) cefazolin, 69.8% (30/43) penicillin G, 58.1% (25/43) ampicillin, 55.8% (24/43) clindamycin, 46.5% (20/43) ceftriaxone, 34.9% (15/43) chloramphenicol, and 30.2% (13/43) for both erythromycin and vancomycin using disk diffusion. Antibiotic resistance genes among the resistant and intermediate-resistant isolates showed high frequencies for *tetM* 97.6% (41/42) and low frequencies for *ermB* 34.5% (10/29), *ermTR* 10.3% (3/29), *mefA* 3.4% (1/29), *tetO* 2.4% (1/42) and *linB* 0% (0/35). The *atr* housekeeping gene yielded 100% (43/43) positive results, whilst the mobile genetic element IS1548 yielded 9.3% (4/43).

**Conclusion:**

The study showed high prevalence of *hly, scpB, bca* and *rib* virulence genes in *S. agalactiae* strains isolated from pregnant women. Tetracycline resistance was predominantly caused by the *tetM* gene, whilst macrolide resistance was predominantly due to the presence of *erm* methylase, with the *ermB* gene being more prevalent. Multi-drug resistance coupled with the recovery of resistant isolates to antimicrobial agents such as penicillins indicates the importance of GBS surveillance and susceptibility tests. It was also observed that in vitro phenotypic resistance is not always accurately predicted by resistance genotypes.

**Supplementary Information:**

The online version contains supplementary material available at 10.1186/s12879-021-05820-6.

## Background

*Streptococcus agalactiae* (Group B *Streptococcus*; GBS) is the leading cause of neonatal infections and serious infections in pregnant women in many countries [1]. GBS generally colonizes asymptomatically as a member of the gastrointestinal or vaginal microbiota but can cause life-threatening infections [2, 3]. About 15 to 35% of pregnant women are colonized vaginally, and approximately 1 to 2% of colonized neonates develop a severe infection [4]. About 25% of pregnant women have *S. agalactiae* in their rectum or vagina [1] which can be passed via vertical transmission to the unborn baby leading to early onset disease in the form of sepsis, pneumonia and meningitis [4].

In low income countries such as Zimbabwe, pregnant women hardly get access to GBS diagnostic tests and antibiotics are not generally affordable for the general public. A study done in three communities in Zimbabwe showed a cumulative 60.3% GBS colonization rate of women who were tested 3 times and that same study showed that GBS colonization is common among pregnant women in the country [5].

*S. agalactiae* uses virulence factors (VFs) encoded by its genes to enter, replicate and persist in a host [6]. Antimicrobial resistance genes confer GBS the ability to resist the effects of medication and these genes are found in the environment [7]. The increasing emergence of antibiotic resistant *S. agalactiae* clearly raises concern for sustained measures in treatment of such life-threatening bacterial infection [8].

The virulence factors in GBS include the capsular polysaccharide antigens (CPA) which is used for strain typing [9, 10]. In Zimbabwe, CPA serotyping and nucleic acid based methods have been used to characterize GBS antigens [11, 12]. Other genes that have been studied in other countries include those for the alpha/α antigens of the C protein (*bca*), beta/β antigens of the C protein (*bac*), surface protein Rib (*rib*), C5a peptidase (*scpB*) and hyaluronate lyase (*hly*) [6, 9, 13]. To our knowledge only one study [11] in this country investigated Rib, Cα and Cβ protein markers in GBS. However, data on some of the other known virulence genes is not yet available.

Penicillin and ampicillin are the drugs of choice for treatment of human *S. agalactiae* infections [14–16] whereas for penicillin-allergic individuals, erythromycin and clindamycin are recommended [1]. Generally, GBS is regarded to be 100% susceptible to penicillin and ampillicin [14, 17, 18]. However, some studies in Ethiopia, USA and Japan have reported clinical isolates exhibiting intermediate and reduced susceptibility to both penicillin G and ampicillin [19–23]. The prevalence of resistance to erythromycin, tetracycline and clindamycin has recently been increasing in *S. agalactiae* [14, 15, 24]. Various researchers have linked this resistance to specific genes found in GBS [14, 16, 25].

Molecular tests are necessary when monitoring the spread of resistant organisms and their genes throughout the communities and medical facilities such as hospitals and clinics [26]. Such tests are important if we are to control the increase in resistance as well as the emergence of multi-drug resistant bacteria. A good example being the study of mobile genetic elements (MGEs) which are a type of DNA that can move around and within the genome [27]. Strategies to fight off bacterial infections by targeting specific virulence genes and mobile genetic elements have been suggested [28].

A total of 13 genes were screened in GBS including 5 virulence genes hyaluronate lyase, *hly*; beta/β antigens of the C protein (*bac*); alpha/α antigens of the C protein (*bca*); surface protein Rib, *rib* [9] and C5a peptidase, *scpB* [29]. The 6 antibiotic resistance genes investigated are the tetracycline resistance determinants *tetM* and *tetO* [25]; erythromycin ribosomal methylase determinants *ermB* and *ermTR*; Macrolide efflux *mefA* and clindamycin resistance determinant *linB* [14]. The Mobile Genetic Element IS1548 [9] and the housekeeping gene *atr* [30] was also amplified. The *atr* gene was selected for analysis in the current study because it is an excellent target for *S. agalactiae* identification. It is an essential gene which has been well studied and has a low probability of mutation [30].

GBS virulence and antibiotic resistance gene studies are important in the development of vaccines and in understanding the resistance mechanisms used by pathogens, respectively. The aim of this study was to identify virulence factors and antibiotic resistance genes found in GBS isolates from pregnant women clinical samples as well as to study the phenotypic antimicrobial resistance and the correlation between the different genes found in GBS isolates.

## Methods

### Study design and sample collection

A cross-sectional study was carried out enrolling 420 healthy pregnant women of 35–38 gestational age on their scheduled Antenatal Care (ANC) routine visit at the Harare and Chitungwiza Central hospitals in Zimbabwe. Using a consecutive sampling method two vaginal swabs were collected during October 2016 to November 2016 period from which the 43 GBS isolates were obtained. Consenting participants were administered with a questionnaire to obtain demographic data, syphilis and HIV status. This was performed by trained research nurses.

### Inclusion and exclusion criteria

Participants who were on treatment for any STI and those who had received antibiotic treatment 1 month prior to recruitment were excluded from the study. All pregnant women who did not give their consent as well as those who were less than 13 weeks pregnant and less than 18 years of age were also excluded from the study.

### Sample size determination

Sample size was calculated using a single proportion estimate with specified precision. A two sided significance level alpha was set at 5%, power of 80% and a prevalence of GBS in Zimbabwe of 31.6% according to Moyo et al., (2000) [31]. The sample size was based on an estimated proportion of 0.33, confidence level of 0.95 and a desired precision of estimate of 0.005. The estimated sample size became 340 plus 5% attrition giving a total minimum sample size of 357.

### Isolation and identification of *Streptococcus agalactiae*

A total of 43 suspected GBS isolates were obtained from the vaginal swabs. These isolates were subcultured by streaking onto 5% sheep blood agar plate (Columbia CNA Agar) (Acumedia, USA). The plates were incubated at 37 °C for 18–24 h in aerobic conditions. The suspected β-hemolytic colonies were identified as GBS using standard microbiological *S. agalactiae* identification tests such as the Gram stain (gram positive cocci), the Catalase test (catalase negative), Bile Aesculin test, CAMP (Christie, Atkins, Munch, Petersen) test which were confirmed using the Streptex grouping latex reagent (Remel, UK). The isolates identified as GBS strains were then stored in a cation-adjusted Mueller Hinton broth along with 20% glycerol at − 80 °C. The negative culture results were issued after 72 h.

### Antimicrobial susceptibility testing

The following 9 antimicrobial discs (Oxoid, Basingstoke, UK) and concentrations were selected: penicillin G (10 units), ampicillin (10 μg), vancomycin (30 μg), cefazolin (30 μg), ceftriaxone (30 μg), chloramphenicol (30 μg), tetracyline (30 μg), clindamycin (2 μg) and erythromycin (15 μg). Antimicrobial susceptibility testing (AST) of GBS was done on 5% sheep blood containing Mueller-Hinton agar using the modified Kirby Bauer methods as recommended by the Clinical Laboratory Standard Institute (CLSI 2017) [32]. The medium was poured into 90 mm diameter sterile petri dishes to a depth of 4 mm (about 25 ml per plate). This was done on a level surface so that the depth of the medium is uniform. Bacterial inocula were prepared by suspending 3–4 freshly grown GBS colonies in 3–5 ml sterile physiological saline and turbidity was adjusted to 0.5 McFarland standard used as a reference to adjust the bacterial suspension. A sterile cotton swab was used to inoculate and excess fluid was removed by pressing and rotating the swab against the side of the tube above the level of the suspension. It was then swabbed over the entire surface of the agar. Then, water-soluble antibiotic impregnated paper disks were placed on the plate and incubated in 5% CO_2_ at 37 °C for 24 h. Incubated, plates were examined to ensure the growth was confluent or near confluent before zone diameters were measured. The minimum inhibitory concentrations (MICs) of ampicillin, chloramphenicol and vancomycin were also confirmed using E-test strips (BioMerieux, Basingstoke, UK). The MICs for three antimicrobials were defined as the lowest concentration of antibiotic that completely inhibited bacterial growth. The susceptibilities of GBS to antibiotics were categorized as sensitive, intermediate and resistant based on the CLSI 2017 guidelines [32].

### Quality control

*Streptococcus pneumoniae* (ATCC 49619), *Streptococcus agalactiae* (ATCC 12386), *Escherichia coli* (ATCC 25922) and *Staphylococcus aureus* (ATCC 29213) were used as quality control in this study.

### DNA isolation and quantification

After subculturing the stored isolates, DNA was extracted using thermal lysis (the boiling method). A pipette was used to place 600 μl of sterile Tris-EDTA (TE) buffer pH 8.0 (Amresco, USA) into a 1.5 ml sterile Eppendorf tube. A sterilized loop was used to emulsify the cultured colonies in TE and this mixture was vortexed for 30–45 s before placing the tightly closed Eppendorf tubes into a boiling water bath for 15 min. A − 20 °C freezer was then used to freeze the boiled mixture for 10 min. DNA samples were stored at − 20 °C in Tris-EDTA buffer at pH 8.0 to minimize the degradation, before being tested for quality and quantity. The preparation of × 1 Tris-Borate electrophoresis (TBE) buffer, 1% agarose gel and molecular weight markers (1 kb and 50 bp) were all done according to manufacturer’s instructions.

The PCR process was done in four stages, i.e. amplification of the housekeeping gene *atr*, the mobile genetic element IS1548, the antibiotic resistance genes (*ermB, ermTR, mefA*, *linB, tetM* and *tetO*) and the virulence genes (*hly, bca, bac, rib* and *scpB*).

### PCR reactions mixtures and conditions

Amplification of all the genes was done using standard PCR. For each DNA sample a 25 μl reaction mixture was made which consisted of 15.875 μl of nuclease-free water (Amresco, USA), 2.5 μl of × 10 PCR buffer (New England BioLabs, UK), 0.5 μl of dNTPs Solution Mix, 10 mM (New England BioLabs, UK), 0.125 μl Taq DNA polymerase, 5 U/ μl (New England BioLabs, UK), 5 μl of DNA template and 1 μl of the 10 μM primer (0.5 μl of forward primer and 0.5 μl of reverse primer) (Inqaba biotech, South Africa). The primers used in our study are listed (see Additional file 2: Table S1). It should be noted that for each PCR test, DNA samples of clinical isolates were simultaneously proliferated and evaluated with a positive control sample (DNA sample of *S. agalactiae*, ATCC 12386) and a negative control (Nuclease free water). A Labnet Multigene OptiMax (Labnet International Inc., USA) was used for the PCR thermal cycling conditions with an initial denaturation step at 94 °C for 1 min, 35 cycles {denaturation 94 °C for 1 min, annealing at 55 °C for 1 min, extension 72 °C for 1 min} and a final elongation step at 72 °C for 10 min. Ten microliters of the amplified products were mixed with 5 μl of gel loading dye (6×) then run along a 1% ethidium bromide (Amresco, USA) (EtBr final conc. of 0.5 μl/ml) stained agarose gel with a 1 kb and 50 bp DNA ladder (New England BioLabs, UK) in 1× TBE buffer (Amresco, USA) for 1 h 30 mins at 100 V (Biobase, China) and then viewed using a Wealtec KETA UV Transilluminator (Wealtec Corp, USA).

The contents of each 25 μl reaction mixture is summarized in Table 1. Optimization of the PCRs was performed by adjusting the DNA template concentration and the annealing time.

**Table 1:** Final primer concentrations and volumes used in the PCRs

Components	25 μl Reaction	Final Conc.
10× PCR Standard Taq reaction buffer	2.5 μl	1×
10 mM dNTPs Solution Mix	0.5 μl	200 μM
10 μM Forward Primer	0.5 μl	0.2 μM
10 μM Reverse Primer	0.5 μl	0.2 μM
Template GBS DNA	5 μl	variable
Taq DNA Polymerase	0.125 μl	1.25 U/50 μl PCR
Nuclease Free Water	15.875 μl	–

### Statistical analysis

All data entry and analysis was done using IBM Statistical Package for the Social Sciences (SPSS) version 24.0 software. Prevalence figures were calculated for the total sample population. Tests for normality were done such as the Shapiro-Wilks test and Skewness-Kurtosis All Normality Test. *P* value less than 0.05 was considered statistically significant.

### Sample characteristics

#### Tetracyline resistance genes

A Shapiro-Wilks test (*p* < 0.05) and a visual inspection of their histograms, normal Q-Q plots and box plots showed that tetracycline resistance was not normally distributed for *tetM* and *tetO* with a skewness of 6.481 (S.E = 0.365) and a kurtosis of 42 (S.E = 6.717) for both *tetM* and *tetO*.

#### Erythromycin resistance genes

A Shapiro-Wilks test (p < 0.05) and a visual inspection of their histograms, normal Q-Q plots and box plots showed that erythromycin resistance was not normally distributed *for ermTR, mefA* and *ermB*, with a skewness of 2 (S.E = 1.014) and a kurtosis of 4 (S.E = 2.679) for *ermTR*, a skewness of 0.816 (S.E = 0.369) and a kurtosis of − 1.405 (S.E = 0.724) for *mefA* and a skewness of 0.321 (S.E = 0.409) and a kurtosis of − 2.023 (S.E = 0.793) for *ermB*.

Clindamycin resistance and the *linB* gene could not be tested for normality because *linB*’s absence was constant and that showed a complete absence of correlation which is represented by 0. The VFs could not also be tested for normality, because they did not have an established dependent and independent variable, however a correlation did exist. Since the data that was tested is not normal, we used non-parametric test, such as the Spearman rank correlation test (see Additional file 2: Table S5). A *p*-value of 0.05 was used for determining statistical significance.

## Results

### Participant characteristics

A total of 420 Zimbabwean pregnant women between 13 and 35 weeks of gestation with a median age of 29 years were recruited and sampled in the study. Of these 10.2% (43/420) were vaginally colonized by GBS and of the 43 women, 95.3% (41/43) were HIV-uninfected and 4.7% (2/43) were HIV-infected.

### Antibiotic susceptibility profiles

The 43 GBS isolates in Table 2 suggest a 97.6% (42/43) resistance of GBS to tetracycline, 72.1% (31/43) cefazolin, 69.8% (30/43) penicillin G, 58.1% (25/43) ampicillin, 55.8% (24/43) clindamycin, 46.5% (20/43) ceftriaxone, 34.9% (15/43) chloramphenicol, and 30.2% (13/43) for both erythromycin and vancomycin.

**Table 2:** Antibiotic susceptibility profiles of 43 GBS isolates from pregnant women

Antibiotic				MIC (μg/ml) ^c^		
	Resistant no. (%)	Intermediate no. (%)	Susceptible no. (%)	MIC_**50**_	MIC_**90**_	Range
**Disk diffusion**^a^						
Penicillin G (10 units)	30/43 (69.8%)	0 (0%)	13/43 (30.2%)	^d^—	–	–
Ampicillin (10 μg)	25/43 (58.1%)	0 (0%)	18/43 (41.9%)	–	–	–
Tetracycline (30 μg)	42/43 (97.6%)	0 (0%)	1 (2.3%)	–	–	–
Clindamycin (2 μg)	24/43 (55.8%)	11/43 (25.6%)	8/43 (18.6%)	–	–	–
Erythromycin (15 μg)	13/43 (30.2%)	16/43 (37.2%)	14/43 (32.6%)	–	–	–
Vancomycin (30 μg)	13/43 (30.2%)	0 (0%)	30/43 (69.8%)	–	–	–
Cefazolin (30 μg)	31/43 (72.1%)	0 (0%)	12/43 (27.9%)	–	–	–
Chloramphenicol (30 μg)	15/43 (34.9%)	16/43 (37.2%)	12/43 (27.9%)	–	–	–
Ceftriaxone (30 μg)	20/43 (46.5%)	0 (0%)	23/43 (53.5%)	–	–	–
**E-test**^b^						
Ampicillin	20/43 (46.5%)	0 (0%)	23/43 (53.5%)	0.25	0.75	0.016 to 4
Vancomycin	16/43 (37.2%)	0 (0%)	30/43 (63.7%)	1	4	0.25 to 8
Chloramphenicol	0/43 (0%)	2/43 (4.7%)	12/43 (95.3%)	1.5	4	0 to 12

^a^ Testing was performed by disk diffusion

^b^ Testing was performed with E-test

^c^MIC_50_ and MIC_90_, MICs at which 50 and 90% of isolates were inhibited, respectively

^d^ —, not available

### Multi-drug resistance pattern

Multidrug resistance (MDR) (≥ 3 drug classes) was detected in 67.4% (29/43) of the isolates. Resistance to 5, 6, 7, 8 and 9 drugs was found to be 34.2%, 17.0%, 23.9%, 20.6% and 3.4% respectively as shown in Table 3.

**Table 3:** Multi-drug resistance pattern of GBS isolated from pregnant women recruited from two health institutions (*n* = 43)

Pattern	Drugs resistance pattern (Antibiogram)	No. of drug to which strains were resistant	No. of MDR strains (%)
I	AMP:PEN:CEFA:VAN:TET	5	3/29 (10.3%)
II	AMP:PEN:CEFA:CLN:TET	5	2/29 (6.9%)
III	AMP:PEN:CEFA:CHL:TET	5	1/29 (3.4%)
IV	AMP:PEN:CLN:CRO:TET	5	1/29 (3.4%)
V	AMP:CEFA:CLN:CRO:TET	5	1/29 (3.4%)
VI	PEN:CEFA:CLN:CRO:TET	5	1/29 (3.4%)
VII	CEFA:ERY:CLN:CHL:TET	5	1/29 (3.4%)
VIII	AMP:PEN:CEFA:ERY:CRO:TET	6	1/29 (3.4%)
IX	AMP:PEN:CEFA:CLN:CRO:TET	6	1/29 (3.4%)
X	PEN:CEFA:CLN:CHL:CRO:TET	6	1/29 (3.4%)
XI	PEN:ERY:CLN:CHL:CRO:TET	6	1/29 (3.4%)
XII	CEFA:ERY:CLN:CHL:CRO:TET	6	1/29 (3.4%)
XIII	AMP:PEN:CEFA:VAN:ERY:CLN:TET	7	3/29 (10.3%)
XIV	AMP:PEN:CEFA:VAN:CLN:CRO:TET	7	1/29 (3.4%)
XV	AMP:PEN:CEFA:ERY:CLN:CRO:TET	7	1/29 (3.4%)
XVI	AMP:PEN:CEFA:VAN:CHL:CRO:TET	7	1/29 (3.4%)
XVII	AMP:PEN:CEFA:CLN:CHL:CRO:TET	7	1/29 (3.4%)
XVIII	AMP:PEN:CEFA:VAN:CLN:CHL:CRO:TET	8	3/29 (10.3%)
XXIV	AMP:PEN:CEFA:ERY:CLN:CHL:CRO:TET	8	2/29 (6.9%)
XXX	AMP:PEN:CEFA:VAN:ERY:CLN:CHL:TET	8	1/29 (3.4%)
XXXI	AMP:PEN:CEFA:VAN:ERY:CLN:CHL:CRO:TET	9	1/29 (3.4%)

*AMP* Ampicillin, *PEN* Penicillin G, *CEFA* Cefazolin, *VAN* Vancomycin, *ERY*, Erythromycin, *CLN* Clindamycin, *CHL* Chloramphenicol, *CRO* Ceftriaxone, *TET* Tetracycline (based on disk diffusion method)

### Virulence gene profiles

Nine distinct virulence gene profiles were identified and the virulence gene profiles *hly-scpB-bca-rib* 37.2% (16/43) and *hly-scpB-bca* 18.6% (8/43) were common among GBS isolates as shown in Table 4.

**Table 4:** Virulence gene profiles of the 43 *Streptococcus agalactiae* isolates

Virulence Gene Profile	Number of Isolates	Frequency %
*hly, bca*	1	2.3
*hly, bca, rib*	3	7.0
*hly, scpB, rib*	6	14.0
*hly, scpB, bca*	8	18.6
*hly, scpB, bca, rib*	16	37.2
*hly, scpB, bca, rib, bac*	1	2.3
*hly, scpB, bca, bac*	4	9.3
*scpB, bca, rib,* IS1548	1	2.3
*hly, scpB, bca, rib,* IS1548	3	7.0

### Prevalence of virulence genes and antibiotic resistance genes

Table 5 shows the percentages of all the genes being investigated (see Additional file 2: Table S2). The housekeeping gene *atr* was present in all 43 isolates, whilst the MGE IS1548 was present in only 4 of the isolates. The virulence gene *hly* had the highest frequency followed by *scpB*, *bca*, *rib* but *bac* which was found in a few isolates. The VFs frequencies were calculated for the whole GBS population (43 isolates). The antibiotic resistance gene *tetM* had a high percentage when compared with *tetO*. The *ermB* determinant was moderately present followed by *ermTR* and *mefA* which was rarely found. The *linB* gene was not detected in any of the isolates. The percentage of the resistant determinants was obtained by combining only the intermediate and resistant populations (I + R) (see Additional file 2: Table S3).

**Table 5:** Frequency of all the 13 genes in the 43 GBS isolates

Name of Gene	Primer Name	Frequency (%)
**Housekeeping Gene**		
	a*tr*	100
**Mobile Genetic Element**		
	IS1548	9.3
**Virulence Genes**		
Hyaluronate lyase	*hly*	97.8
C5a peptidase	*scpB*	90.1
alpha/α antigens of the C protein	*bca*	86.0
Surface protein Rib	*rib*	69.8
beta/β antigens of the C protein	*bac*	11.6
**Antibiotic Resistance Genes**		
Tetracycline Resistance	*tetM*	97.6
Tetracycline Resistance	*tetO*	2.4
Erythromycin Ribosomal Methylase	*ermB*	34.5
Erythromycin Ribosomal Methylase	*ermTR*	10.3
Macrolide efflux	*mefA*	3.4
Clindamycin Resistance	*linB*	0

## Discussion

Our study is the first prevalence report of detection of some of the GBS virulence genes and antibiotic resistance genes in pregnant women in Zimbabwe. The 10.2% prevalence of maternal GBS colonization reported is similar to the 12.2% and 13.4% reported in Ethiopia [19] and Saudi Arabia [33], respectively. However, its lower when compared to the 2010 study in Zimbabwe [5] and this could be due to the effectiveness of some of the ongoing awareness campaigns on pregnant women’s health and early testing. Other possible reasons for these discrepancies are differences in the time of sampling, specimen, sample sizes, geography, detection techniques and gestational ages among other factors.

### The *atr* gene and the Mobile genetic element

The sensitivity of the *atr* gene amplification, in this study was 100% (43/43) (Table 5) which is similar to what is reported in other studies done by Gavino and Wang [34], de-Paris et al.*,* [30] and Alfa et al. [35], where sensitivity was 95.8%, 100% and 90.5% respectively. This high sensitivity may be attributed to the use of selective and enriched media prior to performing PCR. Since this PCR test has shown to be reliable and robust, future studies can focus on rapidly testing pregnant women who present in labor with unknown GBS status. This test was used as a GBS confirmatory identification test. The Mobile Genetic Element IS1548 was present in only 9.3% (4/43) (Table 5). This finding is contrary to an Iranian study which showed that the gene is more prevalent in human than in bovine (77% vs 5%) GBS isolates [9]. The origin of the isolates could be a possible explanation for such a difference.

### Virulence genes

Nine distinct virulence gene profiles were observed but 30/43 strains (69.8%) belonged to *hly-scpB-bca-rib* 37.2% (16/43), *hly-scpB-bca* 18.6% (8/43) or *hly-scpB-rib* 14.0% (6/43) profiles (Table 3). The PCR assay for detection of 'VFs revealed that high percentage of the GBS isolates were positive for *hly* 97.8% (42/43); *scpB* 90.1% (39/43) and *bca* 86.0% (37/43) (Table 5). The high prevalence of these genes in GBS has been previously reported [9, 36–38].

A Zimbabwean study reported a 46.3% of Rib (*rib*) protein marker as well as 19.8% and 15.7% of Cα (*bca*) and Cβ (*bac*) protein markers, respectively [11]. The low frequency obtained for the β protein marker agrees with the 11.6% for *bac* observed in this study and this can be attributed to the reported low distribution of the Ib and II CPA types (11.6% and 8.3%, respectively) in the Zimbabwean GBS population [11]. However, contrary to their findings, the present study reports higher prevalence of *rib* (69.8%) and *bca* (86.0%). This discrepancy could be attributed to differences in CPA types for GBS isolates in these two studies among other factors. Comparisons with this 2008 study, highlighted the importance of investigating the CPA of GBS isolates because a relationship was observed between protein markers and CPA types [11].

Lower frequencies for genes *rib* and *bca*, are reported by Manning et al., (2006) and Persson et al., (2008) with 28% and 43% for *rib* and 29% and 14% for *bca* in the USA [39] and Sweden [40] respectively. However, our higher frequencies agree with the 76.1% for *rib* and 88.6% for *bca* reported in Argentina [41]. The *bac* gene in the present study was found at a similar frequency to the aforementioned studies in USA and Sweden: 11.6% for *bac*, compared with 20% and 12% for *bac*, in the USA and Sweden respectively. The study in Argentina observed a higher frequency of 52.3% for *bac*. A 2017 study in Egypt also observed high frequencies for *scpB* in 100% and *rib* in 79.2% and a lower frequency for *bac* (35.8%) in GBS isolates. However, none of this study’s isolates possessed the *bca* gene [29]. Our comparison with studies from these four countries USA, Sweden, Argentina and Egypt shows that any discrepancies are possibly a result of the geographical locations among other factors.

The results also showed that the majority of the isolates had more than three virulence genes. This high prevalence of various VFs in *S. agalactiae* isolates from the vaginal canal of pregnant women could lead to the development of serious maternal and neonatal infections [42]. However, this study was not able to follow up on the outcomes of both the pregnant mothers and their newborns. The high incidences of VFs except for *bac*, also suggest that GBS vaccines containing the proteins ScpB, α protein [43], Rib [11] and hyaluronate lyase [44] could potentially be effective against our population of pregnant women in Zimbabwe.

The present study observed that all the isolates 100% (*n* = 5) carrying the *bac* gene also carry the unrelated *bca* gene, whilst 86.5% (*n* = 32) of the *bca* carrying isolates did not also have the *bac* gene (see Additional file 2: Table S2). This finding agrees with the report that, isolates that express β tend to also express the unrelated α protein, while the α protein is often expressed on its own in GBS isolates [45, 46]. The majority of the Spearman rank analysis were not statistically significant and with some showing weak associations. However, a significant negative correlation (see Additional file 2: Table S5) was detected between *hly* and IS1548 as well as between *bac* and *rib* genes (*P* < 0.01). This negative association is most likely because these genes are not genetically linked.

### Multi-drug resistance

Multi-drug resistance was shown in 67.4% (29/43) of the isolates with 21 patterns identified (Table 3). Our MDR value is much higher than the 43.9% reported in Ethiopia [21] and yet lower than the 90% reported in GBS in China [47]. Differences in MDR frequencies could be due to use of different antimicrobials, MDR definitions and AST methods. The high resistance observed using disk diffusion method was confirmed by our comparatively higher MIC_50_s and MIC_90_s (Table 2) for ampicillin, vancomycin and chloramphenicol to those reported in a GBS study in Japan [48]. The lowest resistance in our study was observed in 30.2% (13/43) of the GBS isolates to both vancomycin and erythromycin (Table 2). Lower resistance of 17%, 7.5% and 0% to vancomycin, erythromycin and chloramphenicol respectively has been reported by Assefa et al. [21] and other researchers [20, 33]. Higher resistance to chloramphenicol (45%) in GBS isolates has been reported in Iran [15]. Our results showed a 34.9% (15/43) resistance to chloramphenicol using disk diffusion method and 0% (0/43) resistance using the E-test strips (MICs, ≥16 μg/ml). This is because 93.5% (29/31) isolates which had tested intermediate or resistant to chloramphenicol by disk diffusion, were determined to be susceptible by E-test strips (see Additional file 2: Table S4). Both the disk diffusion and E-test results vancomycin showed similar results except for three isolates.

A high resistance to penicillin G 69.8% (30/43) and ampicillin 58.1% (25/43) (disk diffusion method) and 46.5% (20/43) (E-test strips) was observed. This is because five isolates had resistance to ampicillin by disk diffusion but were determined to be susceptible by E-test strips (MICs, ≤0.25 μg/ml). The high resistance is contrary to most studies which report a uniform susceptibility of 100% to all GBS isolates in pregnant women [14, 18, 33, 49, 50]. However, our results agree with recent findings in Ethiopia were a resistance of 77.3% to penicillin G was reported in pregnant women [19]. Other studies have reported resistance to both penicillin G and ampicillin of 10.2% and 9.2% as well as 19.5% and 14.6% respectively [20, 21]. The high resistance reported may be due to the wide and non-prescription use of these two drugs in our study area [49, 51]. It may also be as a result of differences in laboratory procedures or bacterial strains. This study also reports resistance to other penicillin surrogates [52] such as ceftriaxone 46.5% (20/43) and cefazolin 72.1% (31/43). A similar observation of approximately 31% for ceftriaxone was reported [20, 53], whilst a lower range of 0% -15% for cefazolin has been reported [53–55]. We recommend that antibiotic susceptibility testing should be performed if penicillin G or ampicillin therapy is needed in the prevention of neonatal GBS infection. Further studies could focus on identifying the genes responsible for the observed resistance.

### Tetracyline resistance

The high resistance for GBS to tetracycline 97.7% (42/43) (Table 2) can be attributed to a high presence of the *tetM* gene 97.6% (41/42) (see Additional file 1: Fig. 9). This high *tetM* gene presence is as a result of the ubiquitous presence of *tet* genes in pathogens, opportunistic pathogens and members of the normal flora [56]. The high resistance to tetracycline is also because the antibiotic is a relatively cheap, extensively used prophylaxis in the therapy of animal and human infections. It is known that bacterial strains tend to be resistant to frequently used antibiotics [51]. A low presence rate of the *tetO* 2.4% (1/42) (Table 5) indicates that this gene is not common in GBS isolates that are resistant to tetracycline. These results agree with studies done in Canada [57] and Nigeria [58]. In Kuwait, they also reported similar results with 89.5% tetracycline-resistant isolates carrying 94.5% *tetM* and 3.9% *tetO* genes [59].

### Erythromycin and clindamycin resistance

The current study results showed a 30.2% (13/43) and 55.8% (24/43) resistance to erythromycin and clindamycin respectively (Table 2). Such resistance decreases the options of prophylaxis in penicillin allergic women [60]. This combined with the high resistance of GBS to penicillin and ampicillin, we recommend that AST should be performed if clindamycin or erythromycin therapy is needed in the prevention of neonatal GBS infection. Future studies should focus on finding suitable alternative antibiotics for GBS chemoprophylaxis in pregnant women. PCR detected 34.5% (10/29) *ermB*, 10.3% (3/29) *ermTR* and 3.4% (1/29) *mefA* from the intermediate and resistant erythromycin GBS (Table 5). The prevalence of the *ermB* determinant shows that GBS commonly use target methylation as the mechanism of macrolide resistance.

Studies done in Italy [61], South Africa [14], USA [16], Iran [15] and France [25] reported similar findings, with most of the studies also observing that the *ermB* gene is more prevalent in distribution than the *ermTR* gene among GBS strains. This study also confirmed findings by Poyart et al., (2003) that the *mefA* gene is rare among GBS isolates, thus efflux pumps mediated by this gene are not a common mechanism of macrolide resistance [25]. Contrary to Bolukaoto et al., (2015) this current study did not find any *linB* genes 0% (0/35) in any of the clindamycin resistant and intermediate strains [14]. In such cases, where multiple independent resistance genes can cause resistance, the observed phenotypic resistance can be attributed to any of the other known genes or genes that are yet to be discovered. This however limits the usefulness of such diagnostic tests.

It was interesting to note that 63.6% (7/11) GBS which were resistant to both erythromycin and clindamycin had the *ermB* gene (see Additional file 2: Table S2 and S3) and that one GBS strain which was resistant to erythromycin and susceptible to clindamycin carried the *ermTR* gene. Such observations are similar to other studies [16, 62, 63].

### Genetic linkage in macrolide/tetracycline resistance determinant

It was also observed that 100% (*n* = 10) isolates that carried the *ermB* gene also carried the *tetM* gene. The association of erythromycin and tetracycline resistance may be due to the conjugative transposon Tn1545, which encodes erythromycin resistance via the *ermB* gene and tetracycline resistance via the *tetM* gene [64–66]. This association was supported by our analysis which showed a positive correlation (see Additional file 2: Table S5) between the two genes, but this lacked statistical significance according to the Spearman Rank test. Contrary to an Egyptian study [67], we report that there is no evidence suggesting genetic linkage of *tetO* with *ermB, ermTR* or *mefA* in our GBS population.

### Unexpressed resistant genotypes

An unexpected observation in our study was found in the following three (7.0%) isolates. Isolate 127 which was tetracycline sensitive, and yet the *tetM* gene was detected. Isolates 243 and 322, were both erythromycin sensitive however, at least one gene *ermTR* or *mefA* was found, respectively (see Additional file 2: Table S2 and S3). Similar findings were reported in GBS and other streptococcal species [66, 68–70]. However, based on the disk diffusion test, this observation suggests that the Kirby Bauer disk diffusion method is inadequate for detection of resistance. Although the reason(s) behind this lack of gene expression still has to be determined, some possible explanations include gene mutations, absence of selection pressure (i.e. presence of antibiotic), low expression levels of the gene and the possibility of a weak, distant or absent promoter [66, 71, 72]. This study therefore supports the idea that the resistance genotype does not always accurately predict phenotypic resistance.

## Conclusion

An increased resistance against penicillin G, ampicillin, tetracycline, erythromycin, vancomycin and clindamycin among other drugs was observed. The antibiotics erythromycin and vancomycin can be reliable alternatives in treating or preventing GBS infection in women who suffer from colonization or participants that are allergic to penicillin in Harare and Chitungwiza. However, prescribing antibiotic without antibacterial susceptibility tests should be avoided because of the high prevalence of resistance. The tetracycline resistance encoded by ribosome protection gene *tetM* and the ribosomal methylation encoded by erythromycin ribosomal methylase gene, *ermB* were the two most common mechanisms of resistance in GBS from pregnant women. Current study results also showed that resistance genotypes do not always accurately predict phenotypic resistance.

A high frequency of the virulence genes: *hly, scpB, bca* and *rib* was detected in GBS and the majority of the isolates carried multiple virulence genes which showed that GBS’s ability to adhere, colonize, destroy and invade tissues is important in the organism’s pathogenesis. The *bac* gene and the Mobile Genetic Element (IS1548) were rarely found among the GBS isolates from pregnant women.

### Limitations of the study

It would have been necessary to test each isolate using an in vivo animal model, such as mice to demonstrate actual virulence and investigate the expression of resistant genes. A small number of isolates was included from the two centres, which might have caused some bias. Our disc-diffusion assay had no replicates, and this is statistically a weak point. Also pipetting a fixed volume of inoculum for each assay plate is a better method of controlling the volume of inoculum used. In future studies it may be useful to collect information on co-morbidities in participants whose analysis would be related to the various VFs identified.

## Supplementary Information

**Additional file 1.** Gel Electrophoresis Pictograms (Fig. 1 to Fig. 9).

**Additional file 2:** Five supplementary tables; **Table S1.** The oligonucleotide primers used for amplifying various genes in GBS. **Table S2.** Presence or absence of the expected amplicons in the 43 GBS isolates. **Table S3.** Presence or absence of antibiotic resistance genes in the resistant and intermediate isolates. **Table S4.** Disk diffusion zone diameters and E-test results for the 43 GBS isolates. **Table S5.** GBS Virulence and Antibiotic Resistance Genes Spearman Rank Correlations.

## Data Availability

All data generated or analysed during this study are included in this published article [and its supplementary information files].

## Abbreviations

GBS: Group B Streptococcus
VF: virulence factors
CDC: Centers for Disease Control
WHO: World Health Organization
CPA: capsular polysaccharide antigens
MGE: mobile genetic elements
MLSB: Macrolides, Lincosamides, and Streptogramin B
ANC: Antenatal Care
PCR: Polymerase Chain Reaction
AST: Antimicrobial susceptibility testing
