# Supplementary material for: Analysis of virulence factors and antibiotic resistance genes in group B streptococcus from clinical samples
Source: BMC Infect Dis. 2021 Jan 28;21:125. doi: 10.1186/s12879-021-05820-6 (PMC7844887; doi:10.1186/s12879-021-05820-6)
Supplement: Supplementary file 1 — Additional file 1. Gel Electrophoresis Pictograms (Fig. 1 to Fig. 9). [file 12879_2021_5820_MOESM1_ESM.pdf]

Additional file 1

Gel Electrophoresis Pictograms

Amplification of the *atr* housekeeping gene

PCR was done to detect the presence of housekeeping genes. Forty-three genomic DNA samples were amplified using primers targeting for presence of the gene: *atr* (780bp) in GBS. Fig 1 shows the results for the housekeeping gene, *atr*.

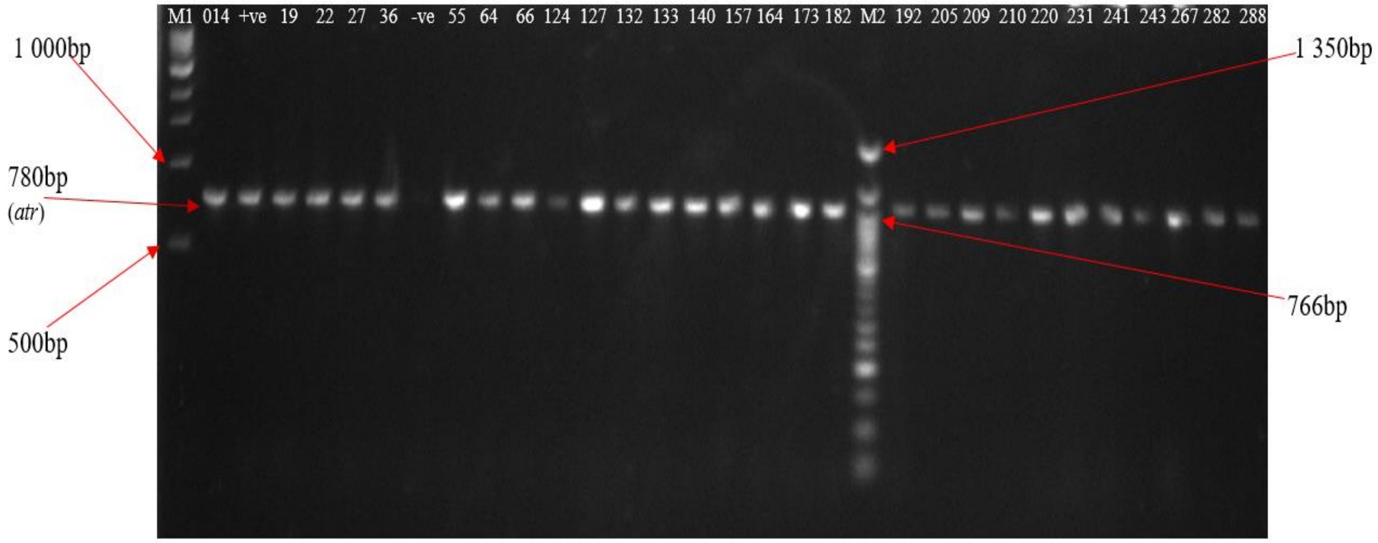

Fig 1: Gel electrophoresis of the *atr* gene. Lane M1 indicates the lane containing the marker, GeneRuler 1 kb Plus DNA Ladder; lane +ve for the positive control; lane -ve for the negative control;; while lane M2 indicates the lane with GeneRuler 50 bp Plus DNA Ladder. : *atr* gene (780 bp) present in all the other 43 GBS isolate.

Amplification of the Mobile Genetic Element, IS1548

PCR was done to detect the presence of MGE. All forty-three genomic DNA samples were amplified using primers targeting for presence of the gene: IS1548 (690 bp) in GBS. Fig 2 shows the results for the MGE, IS1548.

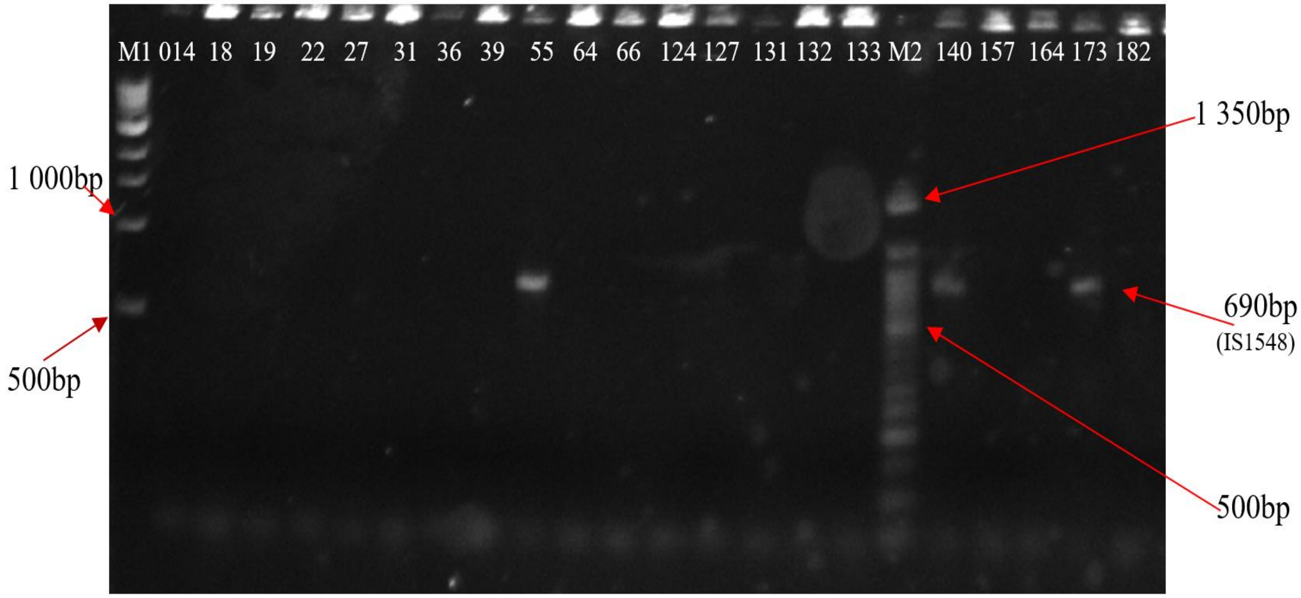

Fig 2: Gel electrophoresis of the Mobile Genetic Element IS1548. Lane M1 indicates the lane containing the marker, GeneRuler 1kb Plus DNA Ladder; while lane M2 indicates the lane with GeneRuler 50 bp Plus DNA Ladder. : IS1548 gene (690 bp) present in GBS isolates 55, 140 and 173 but absent in the rest of the isolates.

Amplification of the genes *atr*, *bac* and *tetO*

All forty-three genomic DNA samples were amplified using PCR primers targeting for presence of the following genes: *atr* (780 bp), *bac* (592 bp) and *tetO* (538 bp) in GBS. Fig 3 shows the results for three genes.

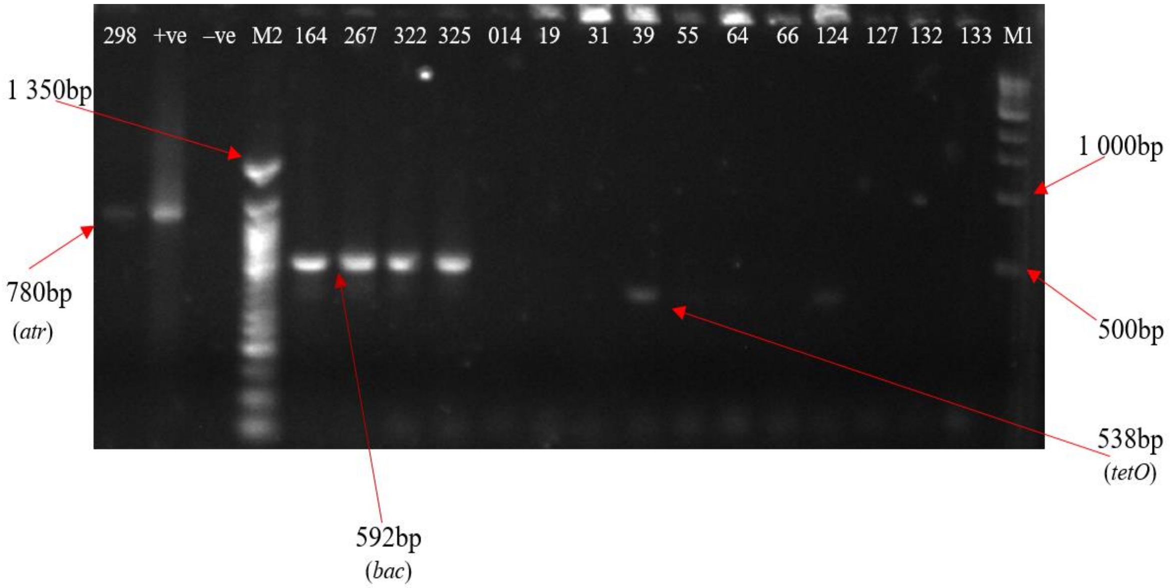

Fig 3: Gel electrophoresis of the *atr*, *bac* and *tetO* genes. Lane M1 indicates the lane containing the marker, GeneRuler 1kb Plus DNA Ladder; while lane M2 indicates the lane with GeneRuler 50 bp Plus DNA Ladder. : *atr* gene (780 bp) present in GBS isolate 298 and the positive control but absent in the negative control. *bac* gene (592 bp) present in GBS isolate 164, 267, 322 and 325. *tetO* gene (538 bp) present in GBS isolates 39 and 124 but absent in isolates 014, 19, 31, 55, 64, 66, 127, 132 and 133.

Amplification of the genes *scpB, ermB* and *rib*

All forty-three genomic DNA samples were amplified using PCR primers targeting for presence of the following genes: *scpB* (255 bp), *ermB* (640 bp) and *rib* (369 bp) in GBS. Fig 4 shows the results for three genes.

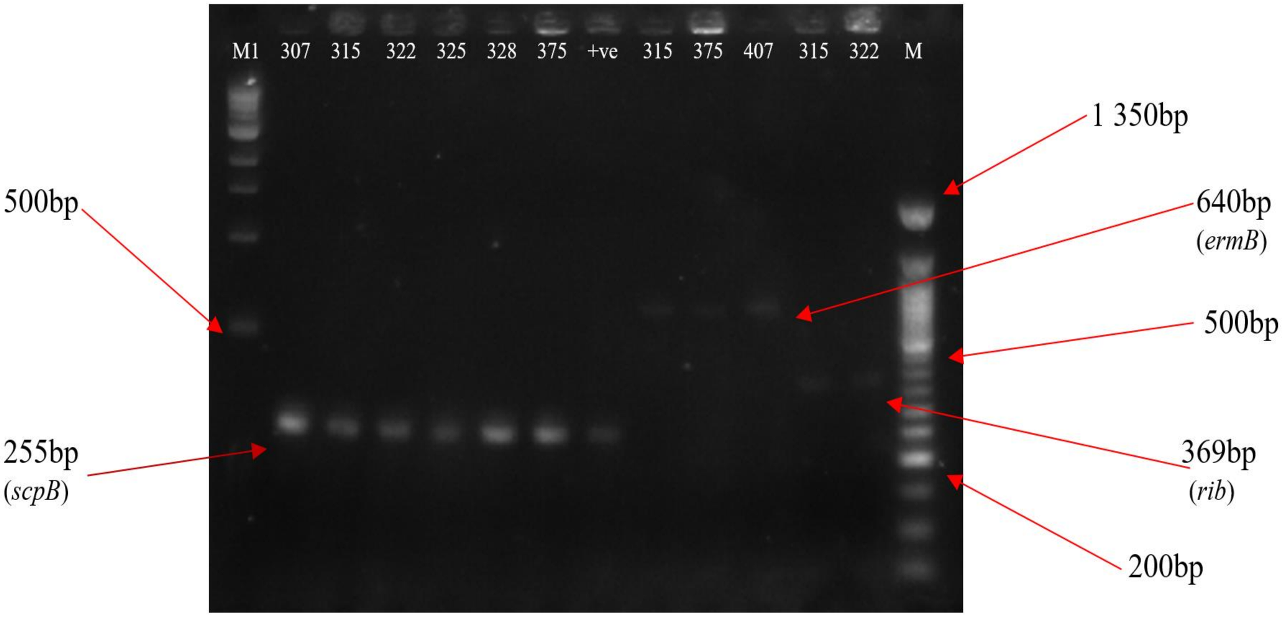

Fig 4: Gel electrophoresis of the *scpB, ermB* and *rib* genes. Lane M1 indicates the lane containing the marker, GeneRuler 1 kb Plus DNA Ladder; lane +ve for the positive control; while lane M2 indicates the lane with GeneRuler 50 bp Plus DNA Ladder. : *scpB* (255 bp) present in GBS isolates 307, 315, 322, 325, 328, 375 and the positive control. *ermB* (640 bp) present in GBS isolates 315, 375 and 407. *rib* (369 bp) present in GBS isolates 315 and 322.

Amplification of the genes *linB, rib, hly* and *scpB*

All forty-three genomic DNA samples were amplified using PCR primers targeting for presence of the following genes: *linB* (944 bp), *rib* (369 bp), *hly* (199 bp) and *scpB* (255 bp) in GBS. Fig 5 shows the results for three genes.

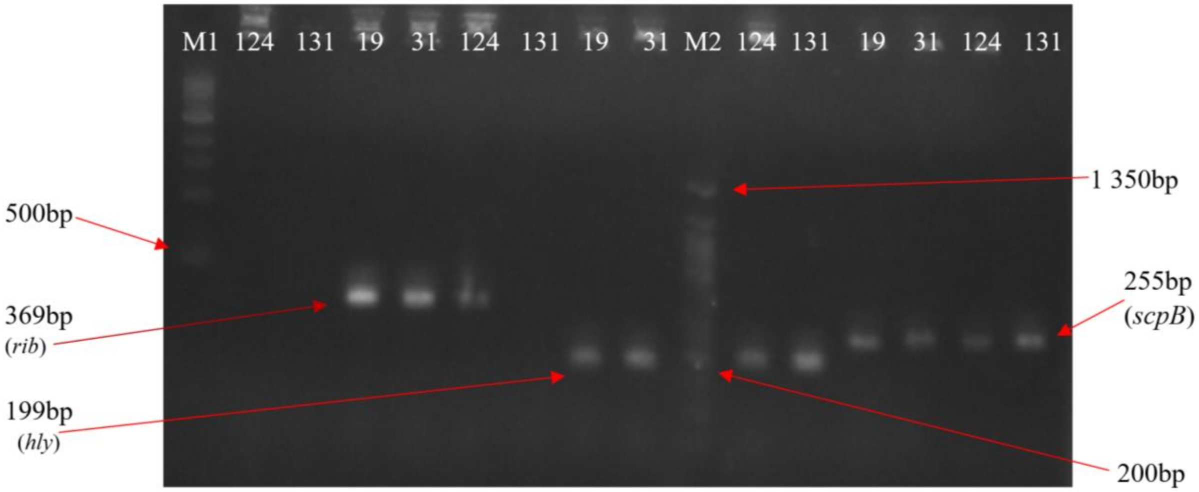

Fig 5: Gel electrophoresis of the *linB*, *rib*, *hly* and *scpB* genes. Lane M1 indicates the lane containing the marker, GeneRuler 1kb Plus DNA Ladder; while lane M2 indicates the lane with GeneRuler 50 bp Plus DNA Ladder. : *linB* (944 bp) absent in GBS isolate 124 and 131. *rib* (369 bp) present in GBS isolates 19, 31, 124 but absent in 131. *hly* (199 bp) present in GBS isolates 19, 31,124 and 131. *scpB* (255 bp) present in GBS isolates19,31,124 and 131.

Amplification of the gene *bca*

All forty-three genomic DNA samples were amplified using PCR primers targeting for presence of the virulence gene: *bca* (535 bp) in GBS. Fig 6 shows the results for the *bca* gene.

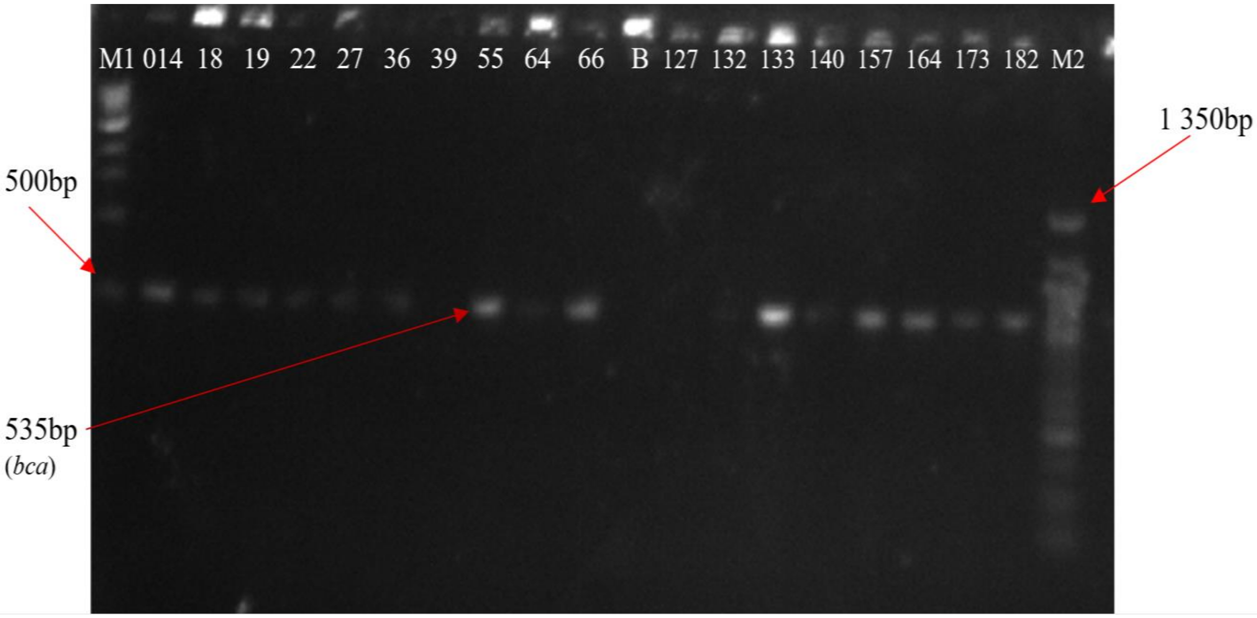

Fig 6: Gel electrophoresis of the *bca* gene. Lane M1 indicates the lane containing the marker, GeneRuler 1 kb Plus DNA Ladder; lane B for the blank/negative control; while lane M2 indicates the lane with GeneRuler 50 bp Plus DNA Ladder. : *bca* (535 bp) absent in GBS isolate B(blank), 39, 127 and 131.

Amplification of the genes *bac, mefA* and *tetO*

All forty-three genomic DNA samples were amplified using PCR primers targeting for presence of the following genes: *bac* (592 bp), *mefA* (348 bp) and *tetO* (538 bp) in GBS. Fig 7 shows the results for three genes.

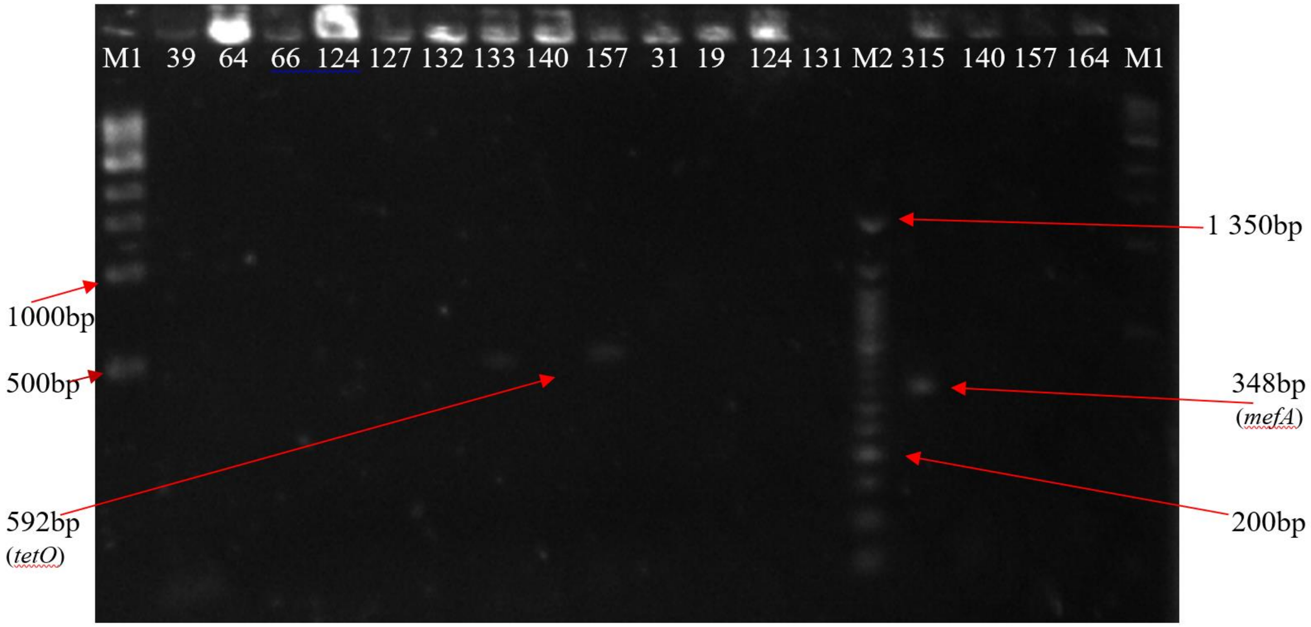

Fig 7: Gel electrophoresis of the *bac, mefA* and *tetO* genes. Lane M1 indicates the lane containing the marker, GeneRuler 1 kb Plus DNA Ladder; while lane M2 indicates the lane with GeneRuler 50 bp Plus DNA Ladder. : *bac* (592 bp) present in GBS isolate 133 and 157 but absent in GBS isolates 39, 64, 66, 124, 127, 132, 140, 31, 19, 124 and 131. *mefA* gene (348 bp) present in GBS isolate 315. *tetO* (538 bp) absent in GBS isolates 140, 157 and 164.

Amplification of the genes *ermTR* and *bac*

All forty-three genomic DNA samples were amplified using PCR primers targeting for presence of the following genes: *ermTR* (400 bp) and *bac* (592 bp) in GBS. Fig 8 shows the results for two genes.

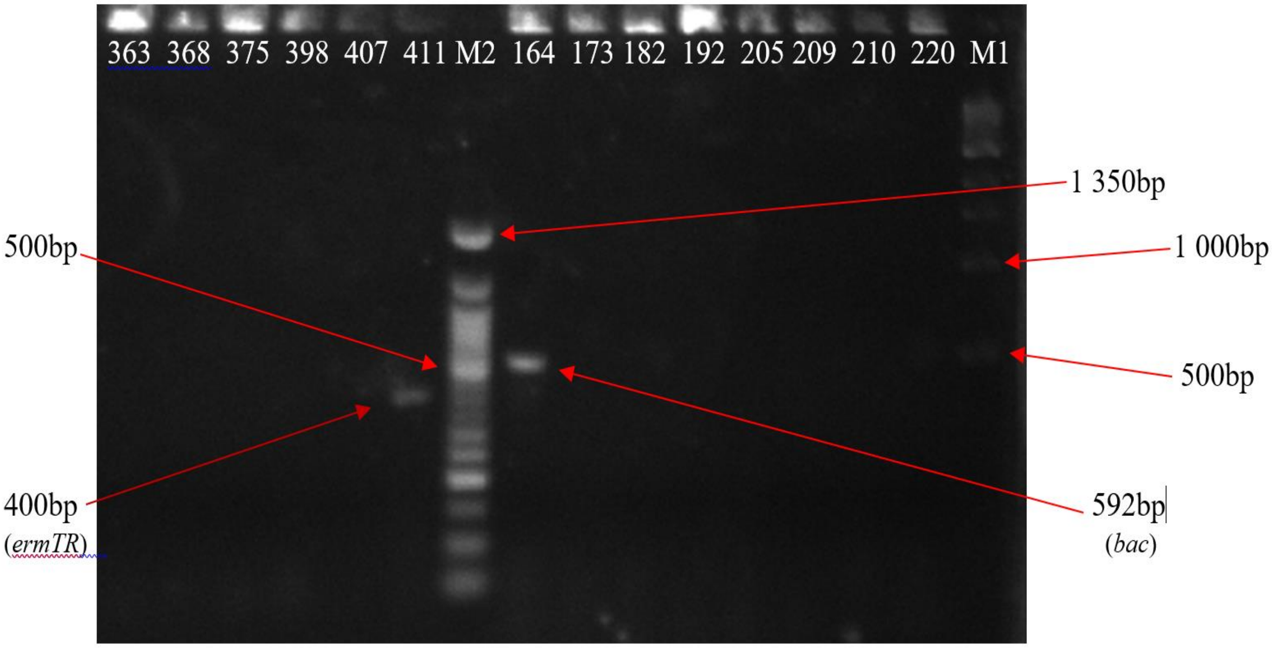

Fig 8: Gel electrophoresis of the *ermTR* and *bac* genes. Lane M1 indicates the lane containing the marker, GeneRuler 1 kb Plus DNA Ladder; while lane M2 indicates the lane with GeneRuler 50 bp Plus DNA Ladder. : *ermTR* gene (400 bp) present in GBS isolate 411 but absent in 363, 368, 375 and 407. *bac* (592 bp) present in GBS isolate 164 but absent in GBS isolates 173, 182, 192,205, 209, 210 and 220.

Amplification of the gene *tetM*

All forty-three genomic DNA samples were amplified using PCR primers targeting for presence of the following antibiotic resistance gene: *tetM* (359 bp) in GBS. Fig 9 shows the results for one gene.

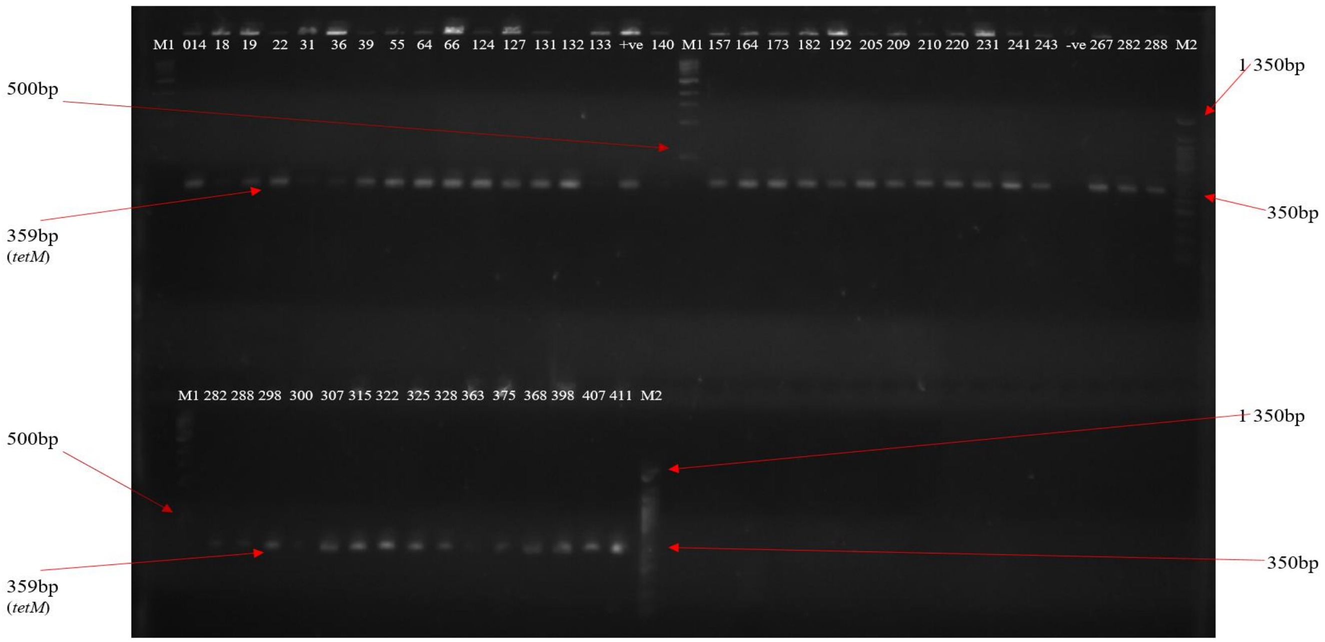

Fig 9: Gel electrophoresis of the *tetM* gene. Lane M1 indicates the lane containing the marker, GeneRuler 1 kb Plus DNA Ladder; lane +ve for the positive control; lane -ve for the negative control; while lane M2 indicates the lane with GeneRuler 50 bp Plus DNA Ladder. : *tetM* (359 bp) present in all the other 43 GBS isolates except for sample 140
