# Supplementary material for: Analysis of virulence factors and antibiotic resistance genes in group B streptococcus from clinical samples
Source: BMC Infect Dis. 2021 Jan 28;21:125. doi: 10.1186/s12879-021-05820-6 (PMC7844887; doi:10.1186/s12879-021-05820-6)
Supplement: Supplementary file 2 — Additional file 2: Five supplementary tables; Table S1. The oligonucleotide primers used for amplifying various genes in GBS. Table S2. Presence or absence of the expected amplicons in the 43 GBS isolates. Table S3. Presence or absence of antibiotic resistance genes in the resistant and intermediate isolates. Table S4. Disk diffusion zone diameters and E-test results for the 43 GBS isolates. Table S5. GBS Virulence and Antibiotic Resistance Genes Spearman Rank Correlations. [file 12879_2021_5820_MOESM2_ESM.pdf]

Additional File 2

All the genes discussed in this article are all summarized below (Table S1).

Table S1: The oligonucleotide primers used for amplifying various genes in GBS

Primer Name	Name of Gene	Primer sequence (5' to 3')	Expected Product size	Reference
atr		F- CAA CGA TTC TCT CAG CTT TGT TAA<br>R- TAA GAA ATC TCT TGT GCG GAT TTC	780	(30)
IS1548		F- TTG CGC AGT TGA ATT GGA TAG<br>R- TTC TCT AAC TTC AAT CTG TCC CCT A	690	(9)
bac	beta/β antigens of the C protein	F- CTA TTT TTG ATA TTG ACA ATG CAA<br>R- GTC GTT ACT TCC TTG AGA TGT AAC	592	(9)
bca	alpha/α antigens of the C protein	F- TAA CAG TTA TGA TAC TTC ACA GAC<br>R- ACG ACT TTC TTC CGT CCA CTT AGG	535	(9)
hly	Hyaluronate lyase	F- TCC ATT TAA AGC CCT TGG TG<br>R- GGC GCC AGT ATA AGC AAC AT	199	(9)
rib	Surface protein Rib	F- CAG GAA GTG CTG TTA CGT TAA AC<br>R- CGT CCC ATT TAG GGT CTT CC	369	(9)
scpB	C5a peptidase	F- ACA ACG GAA GGC GCT ACT GTT C<br>R- ACC TGG TGT TTG ACC TGA ACT A	255	(29)
ermB	Erythromycin Ribosomal Methylase	F- GAA AAG GTA CTC AAC CAA ATA<br>R- AGT AAC GGT ACT TAA ATT GTT TAC	640	(14)
ermTR	Erythromycin Ribosomal Methylase	F- GAA GTT TAG CTT TCC TAA<br>R- GCT TCA GCA CCT GTC TTA ATT GAT	400	(14)
mefA	Erythromycin resistance efflux pump	F- AGT ATC ATT AAT CAC TAG TGC<br>R- TTC TTC TGG TAC TAA AAG TGG	348	(14)
linB	Lincosamide nucleotidyltransferases	F- CCT ACC TAT TGT TTG TGG AA<br>R- ATA ACG TTA CTC TCC TAT TC	944	(14)
tetM	Tetracycline Resistance	F- GTG GAG TAC TAC ATT TAC GAG<br>R- GAA GCG GAT CAC TAT CTG AG	359	(25)
tetO	Tetracycline Resistance	F- GCG GAA CAT TGC ATT TGA GGG<br>R- CTC TAT GGA CAA CCC GAC AGA AG	538	(25)

All the primers used in this study were from Inqaba biotech, South Africa

Table S2: Presence or absence of the expected amplicons in the 43 GBS isolates

No.	Sample #	Housekeeping gene	MGE	Virulence Genes					Antibiotic Resistance Genes					
		atr	**IS1548**	***rib***	***hly***	***scpB***	***bca***	***bac***	***tetM***	***tetO***	***ermB***	***ermTR***	***mefA***	***linB***
**1**	**014**	+	_	_	+	+	+	_	+	_	_	_	_	_
**2**	**19**	+	_	+	+	+	+	_	+	_	_	+	_	_
**3**	**22**	+	_	+	+	_	+	_	+	_	_	_	_	_
**4**	**27**	+	_	+	+	+	+	_	+	_	_	_	_	_
**5**	**31**	+	_	+	+	+	+	_	+	_	+	_	_	_
**6**	**36**	+	_	+	+	+	+	_	+	_	_	_	_	_
**7**	**55**	+	+	+	_	+	+	_	+	_	_	_	_	_
**8**	**64**	+	_	+	+	+	+	_	+	_	_	_	_	_
**9**	**66**	+	_	+	+	+	+	_	+	_	_	_	_	_
**10**	**124**	+	_	+	+	+	+	_	+	+	_	_	_	_
**11**	**127**	+	_	+	+	+	_	_	+	_	_	_	_	_
**12**	**131**	+	_	_	+	+	+	_	+	_	+	_	_	_
**13**	**132**	+	_	+	+	_	+	_	+	_	_	_	_	_
**14**	**133**	+	_	_	+	_	+	_	+	_	_	_	_	_
**15**	**140**	+	+	+	+	+	+	_	_	_	_	_	_	_
**16**	**157**	+	_	_	+	+	+	+	+	_	_	_	_	_
**17**	**164**	+	_	_	+	+	+	+	+	_	+	_	_	_
**18**	**173**	+	+	+	+	+	+	_	+	_	_	_	_	_
**19**	**182**	+	_	+	+	+	+	_	+	_	_	_	_	_
**20**	**192**	+	_	+	+	+	_	_	+	_	+	_	_	_
**21**	**205**	+	_	+	+	+	_	_	+	_	_	+	_	_
**22**	**209**	+	_	+	+	+	_	_	+	_	_	_	_	_
**23**	**210**	+	_	+	+	+	+	_	+	_	_	_	_	_
**24**	**220**	+	_	+	+	+	+	_	+	_	_	_	_	_
**25**	**231**	+	_	+	+	+	+	_	+	_	_	_	_	_
**26**	**241**	+	_	+	+	+	+	_	+	_	_	_	_	_
**27**	**243**	+	_	+	+	+	_	_	+	_	_	+	_	_
**28**	**267**	+	_	_	+	+	+	+	+	_	_	_	_	_
**29**	**282**	+	_	+	+	+	+	_	+	_	_	_	_	_
**30**	**288**	+	_	_	+	+	+	_	+	_	_	_	_	_
**31**	**298**	+	_	+	+	+	+	_	+	_	_	_	_	_
**32**	**300**	+	_	+	+	_	+	_	+	_	_	_	_	_
**33**	**307**	+	_	+	+	+	+	_	+	_	_	_	_	_
**34**	**315**	+	+	+	+	+	+	_	+	_	+	_	+	_
**35**	**322**	+	_	+	+	+	+	+	+	_	_	_	+	_
**36**	**325**	+	_	_	+	+	+	+	+	_	+	_	_	_
**37**	**328**	+	_	_	+	+	+	_	+	_	_	_	_	_
**38**	**363**	+	_	+	+	+	_	_	+	_	+	_	_	_
**39**	**368**	+	_	_	+	+	+	_	+	_	_	_	_	_
**40**	**375**	+	_	+	+	+	+	_	+	_	+	_	_	_
**41**	**398**	+	_	_	+	+	+	_	+	_	+	_	_	_
**42**	**407**	+	_	_	+	+	+	_	+	_	+	_	_	_
**43**	**411**	+	_	_	+	+	+	_	+	_	_	+	_	_

Presence (+) or absence (-) of the expected amplicon during the PCRs.

Table S3: Presence or absence of antibiotic resistance genes in the resistant and intermediate isolates

Number	Sample #	CLN (2 µg)	*linB*	ERY (15 µg)	*ermB*	*ermTR*	*mefA*	TET (30 µg)	*tetM*	*tetO*
**1**	**014**	R	_	I	_	_	_	R	+	_
**2**	**19**			R	_	+	_	R	+	_
**3**	**22**	R	_					R	+	_
**4**	**27**	R	_	I	_	_	_	R	+	_
**5**	**31**	R	_	R	+	_	_	R	+	_
**6**	**36**	R	_	R	_	_	_	R	+	_
**7**	**55**	R	_	I	_	_	_	R	+	_
**8**	**64**	R	_	R	_	_	_	R	+	_
**9**	**66**	I	_	I	_	_	_	R	+	_
**10**	**124**	R	_	I	_	_	_	R	+	+
**11**	**127**	I	_							
**12**	**131**	R	_	R	+	_	_	R	+	_
**13**	**132**							R	+	_
**14**	**133**							R	+	_
**15**	**140**	I	_	I	_	_	_	R	_	_
**16**	**157**	I	_					R	+	_
**17**	**164**	R	_	R	+	_	_	R	+	_
**18**	**173**	R	_	R	_	_	_	R	+	_
**19**	**182**							R	+	_
**20**	**192**	R	_	R	+	_	_	R	+	_
**21**	**205**	I	_	R	_	+	_	R	+	_
**22**	**209**	I	_	I	_	_	_	R	+	_
**23**	**210**	R	_					R	+	_
**24**	**220**	I	_					R	+	_
**25**	**231**	R	_	I	_	_	_	R	+	_
**26**	**241**	R	_	I	_	_	_	R	+	_
**27**	**243**							R	+	_
**28**	**267**							R	+	_
**29**	**282**	I	_					R	+	_
**30**	**288**	R	_					R	+	_
**31**	**298**			I	_	_	_	R	+	_
**32**	**300**	R	_	R	_	_	_	R	+	_
**33**	**307**	R	_	I	_	_	_	R	+	_
**34**	**315**	R	_	R	+	_	+	R	+	_
**35**	**322**							R	+	_
**36**	**325**	R	_	I	+	_	_	R	+	_
**37**	**328**	I	_					R	+	_
**38**	**363**	R	_	R	+	_	_	R	+	_
**39**	**368**	I	_	I	_	_	_	R	+	_
**40**	**375**	R	_	R	+	_	_	R	+	_
**41**	**398**	R	_	I	+	_	_	R	+	_
**42**	**407**	I	_	I	+	_	_	R	+	_
**43**	**411**	R	_	I	_	+	_	R	+	_

Presence (+) or absence (-) of the expected amplicon during the PCRs.

Table S4: Disk diffusion zone diameters and E-test results for the 43 GBS isolates

	Disk Diffusion (mm)									E-test 0.006-256 (μg/ml)		
Sample #	**10 μg AMP**	**10 Units PEN**	**30 μg CEFA**	**30 μg VAN**	**15 μg ERY**	**2 μg CLN**	**30 μg CHL**	**30 μg CRO**	**30 μg TET**	**E-AMP**	**E-VAN**	**E-CHL**
**14**	R (21)	S (28)	R (12)	S (17)	I (17)	R (12)	I (19)	R (23)	R (0)	R (0.75)	R (3)	S (1.5)
**19**	S (26)	S (28)	S (30)	S (18)	R (0)	S (19)	I (20)	S (27)	R (0)	S (0.064)	S (0.75)	S (2)
**22**	R (22)	R (20)	R (12)	S (17)	S (22)	R (12)	S (22)	S (26)	R (0)	R (0.75)	R (4)	S (1.5)
**27**	R (20)	R (17)	R (12)	R 14)	I (20)	R (0)	I (18)	R (0)	R (0)	R (0.75)	R (3)	I (12)
**31**	R (18)	R (15)	R (12)	S (20)	R (0)	R (0)	S (22)	R (16)	R (0)	R (0.50)	S (1.0)	S (2)
**36**	R (20)	R (22)	R (11)	R (16)	R (8)	R (0)	S (22)	S (25)	R (0)	R (0.50)	R (4)	S (4)
**55**	R (21)	R (16)	R (12)	S (17)	I (16)	R (0)	S (23)	S (30)	R (0)	R (0.50)	S (1.0)	S (0.38)
**64**	R (20)	R (17)	R (10)	S (17)	R (13)	R (0)	R (0)	R (0)	R (0)	R (0.50)	R (4)	S (1.5)
**66**	R (18)	R (17)	R (0)	R (16)	I (16)	I (16)	R (16)	R (21)	R (0)	R (0.50)	S (1.0)	S (1.0)
**124**	S (25)	R (18)	R (12)	S (19)	I (16)	R (0)	I (19)	R (14)	R (5)	S (0.25)	R (2)	S (1.5)
**127**	S (26)	S (24)	S (27)	S (18)	S (21)	I (18)	S (22)	S (30)	S (28)	S (0.064)	S (0.38)	S (1.5)
**131**	R (23)	R (17)	R (11)	R (15)	R (0)	R (0)	I (18)	S (24)	R (0)	R (0.75)	R (3)	S (0.38)
**132**	S (26)	R (21)	S (25)	S (20)	S (25)	S (24)	S (25)	S (30)	R (0)	S (0.094)	S (1.0)	S (0.50)
**133**	R (0)	R (0)	R (10)	R (16)	S (23)	S (20)	S (24)	S (27)	R (13)	S (0.25)	R (1.5)	S (1.0)
**140**	S (26)	S (27)	S (27)	S (17)	I (17)	I (17)	R (17)	S (25)	R (12)	S (0.023)	S (0.75)	S (2)
**157**	S (25)	S (27)	S (27)	S (17)	S (22)	I (18)	I (18)	R (22)	R (0)	S (0.032)	S (1.0)	S (1.0)
**164**	R (18)	R (17)	R (12)	R (16)	R (0)	R (0)	I (18)	S (30)	R (10)	R (0.50)	R (2)	S (1.5)
**173**	R (21)	R (20)	R (13)	S (18)	R (15)	R (15)	R (17)	R 23	R (7)	R (0.75)	S (0.25)	S (0.50)
**182**	S (29)	S (30)	S (24)	S (19)	S (26)	S (20)	I (20)	S (24)	R (0)	S (0.016)	S (0.38)	S (4)
**192**	R (23)	R (17)	R (12)	R (16)	R (8)	R (0)	R (8)	S (26)	R (0)	R (0.75)	R (1.5)	S (2)
**205**	R (22)	R (16)	R (11)	S (18)	R (10)	I (17)	I (19)	R (23)	R (9)	S (0.064)	S (1.0)	S (1.5)
**209**	R (22)	R (18)	R (10)	S (18)	I (20)	I (18)	I (20)	S (30)	R (0)	R (0.75)	S (1.0)	S (1.5)
**210**	S (29)	S (29)	S (28)	S (17)	S (21)	R (15)	S (26)	S (26)	R (6)	S (0.023)	S (0.38)	S (1.5)
**220**	R (20)	R (15)	R (11)	S (17)	S (24)	I (18)	R (16)	S (27)	R (0)	R (0.75)	S (1.0)	S (0)
**231**	R (16)	R (15)	R (8)	R (14)	I (17)	R (0)	R (13)	R (0)	R (0)	R (0.75)	R (4)	I (12)
**241**	S (25)	R (20)	R (12)	S (17)	I (18)	R (0)	R (17)	R (0)	R (0)	S (0.064)	S (0.50)	S (2)
**243**	S (30)	S (29)	S (29)	S (19)	S (22)	S (19)	I (20)	S (30)	R (0)	S (0.047)	S (0.38)	S (1.5)

**267**	S (30)	S (28)	S (27)	S (25)	S (22)	S (24)	S (23)	S (24)	R (6)	S (0.016)	S (0.25)	S (1.5)
**282**	R (17)	R (17)	R (14)	R (16)	S (25)	I (18)	I (20)	S (26)	R (0)	R (1.0)	R (4)	S (4)
**288**	R (20)	R (15)	R (11)	S (18)	S (25)	R (9)	S (23)	R (22)	R (12)	S (0.12)	S (0.25)	S (0.38)
**298**	S (25)	R (23)	R (22)	S (18)	I (20)	S (22)	I (20)	S (27)	R (0)	R (0.5)	S (0.38)	S (1.0)
**300**	S (24)	R (23)	S (29)	S (18)	R (15)	R (15)	R (15)	R (23)	R (0)	S (0.064)	S (0.75)	S (4)
**307**	R (22)	R (16)	R (10)	R (16)	I (20)	R (15)	R (17)	R (21)	R (0)	R (0.75)	R (8)	S (2)
**315**	S (24)	S (25)	R (21)	S (18)	R (12)	R (15)	R (15)	R (17)	R (0)	S (0.064)	S (0.38)	S (2)
**322**	S (27)	S (26)	R (14)	S (17)	S (21)	S (20)	S (22)	S (30)	R (12)	S (0.19)	S (0.75)	S (0.75)
**325**	R (21)	R (17)	R (12)	S (17)	I (16)	R (0)	R (17)	R (0)	R (0)	S (0.064)	S (0.75)	S (1.0)
**328**	S (27)	S (25)	S (27)	S (18)	S (22)	I (17)	S (23)	R (22)	R (14)	S (0.047)	S (1.0)	S (1.5)
**363**	R (22)	R (17)	R (8)	R (16)	R (0)	R (0)	R (0)	R (15)	R (0)	S (0.125)	R (2)	S (0.125)
**368**	R (20)	R (18)	R (12)	R (16)	I (20)	I (17)	I (20)	S (25)	R (0)	R (0.75)	R (2)	S (1.5)
**375**	S (30)	S (28)	R (18)	S (18)	R (0)	R (0)	R (0)	S (24)	R (0)	S (0.064)	S (0.75)	S (3)
**398**	R (21)	R (16)	R (8)	R (10)	I (16)	R (0)	R (17)	R (0)	R (0)	R (4)	R (1.5)	S (1.5)
**407**	S (25)	R (14)	R (8)	S (17)	I (20)	I (18)	I (18)	S (24)	R (0)	S (0.19)	S (1.0)	S (0.25)
**411**	R (20)	R (23)	S (24)	S (18)	I (16)	R (14)	I (18)	R (22)	R (0)	S (0.04)	S (0.75)	S (4)
**CLSI 2017 ranges**	≥24 (S)	≥24 (S)	≥24 (S)	≥17 (S)	≥21 (S), 16-20 (I), ≤15 (R)	≥19 (S), 16-18 (I), ≤15 (R)	≥21 (S), 18-20 (I), ≤17 (R)	≥24 (S)	≥23 (S), 19-22 (I), ≤18 (R)	≤0.25 (S)	≤1 (S)	≤4 (S), 8 (I), ≥16 (R)
***S.pneumonia* ATCC 49619**	30	30	30	20	28	22	22	35	22	0.025	0.25	2
**Control Ranges and Comment**	30-36 Pass	24-30 Pass		20-27 Pass	25-30 Pass	19-25 Pass	23-27 Pass	30-35 Pass	27-31 Pass	0.06-0.25 Pass	0.12-0.5 Pass	2-8 Pass

Spearman Rank Correlations

Table S4 below shows that there was a negative correlation between *tetO* and all the macrolide resistant determinants; between *ermB* and *ermTR* and also between *ermTR* and *mefA*. A positive correlation was observed between the *tetM* and all the macrolide resistant determinants (such as *ermB*); between *tetM* and *tetO* and also between *ermB* and *mefA*. However, the P values for all these correlations are greater than the level of significance implying that correlations are not statistically significant, meaning they have occurred by chance. Hence, they cannot be generalized into the general GBS population. The *linB* gene could not be computed because it was a constant. Table 3 also shows that there is a negative correlation between *hly* and IS1548 as well as between *bac* and *rib* and this is statistically significant at ($p < 0.01$). These relationships can be generalized into the pregnant women GBS population. However, some non-statistically significant positive correlations were also observed between IS1548 and (*rib/scpB/bca*) as well as *bac* and (*hly/scpB/bca*). The remainder of the genes had a negative correlation which was also not statistically significant.

Table S5: GBS Virulence and Antibiotic Resistance Genes Spearman Rank Correlations

Virulence Genes	IS1548	*rib*	*hly*	*scpB*	*bca*	*bac*
**IS1548**	1	0.18	-.563**	0.088	0.11	-0.099
rib	0.18	1	-0.102	-0.036	-0.265	-.393**
hly	-.563**	-0.102	1	-0.049	-0.062	0.056
scpB	0.088	-0.036	-0.049	1	-0.129	0.116
bca	0.11	-0.265	-0.062	-0.129	1	0.146
bac	-0.099	-.393**	0.056	0.116	0.146	1
**Antibiotic Resistance Genes**	***tetM***	***tetO***	***ermB***	***ermTR***	***mefA***	***linB***
tetM	1	0.024	0.085	0.049	0.034	.a
tetO	0.024	1	-0.085	-0.049	-0.034	.a
ermB	0.085	-0.085	1	-0.176	0.14	.a
ermTR	0.049	-0.049	-0.176	1	-0.071	.a
mefA	0.034	-0.034	0.14	-0.071	1	.a
linB	.a	.a	.a	.a	.a	.a

* Correlation is significant at the 0.05 level (2-tailed).
** Correlation is significant at the 0.01 level (2-tailed).
a Cannot be computed because at least one of the variables is constant.
